# A thermosensitive, reactive oxygen species-responsive, MR409-encapsulated hydrogel ameliorates disc degeneration in rats by inhibiting the secretory autophagy pathway

**DOI:** 10.7150/thno.47723

**Published:** 2021-01-01

**Authors:** Qiangqiang Zheng, Haotian Shen, Zongrui Tong, Linxiang Cheng, Yuzi Xu, Zhiyun Feng, Shiyao Liao, Xiaojian Hu, Zongyou Pan, Zhengwei Mao, Yue Wang

**Affiliations:** 1 Spine lab, Department of Orthopedic Surgery, The First Affiliated Hospital, Zhejiang University School of Medicine, Hangzhou 310003, China.; 2 MOE Key Laboratory of Macromolecular Synthesis and Functionalization, Department of Polymer Science and Engineering, Zhejiang University, Hangzhou 310027, China.; 3 Department of Orthopedic Surgery, Zhejiang Provincial People's Hospital, Hangzhou Medical College, Hangzhou 310003, China.; 4 Department of Oral Implantology and Prosthodontics, The Affiliated Stomatology Hospital, Zhejiang University School of Medicine, Hangzhou, 310006, P.R. China.; 5 Dr. Li Dak Sum & Yip Yio Chin Center for Stem Cells and Regenerative Medicine, And Department of Orthopedic Surgery of the Second Affiliated Hospital, Zhejiang University School of Medicine, Hangzhou, China.

**Keywords:** disc degeneration, secretory autophagy, MR409, ROS responsive carrier, injectable hydrogel

## Abstract

Lumbar disc degeneration is a common cause of chronic low back pain and an important contributor to various degenerative lumbar spinal disorders. However, currently there is currently no effective therapeutic strategy for treating disc degeneration. The pro-inflammatory cytokine interleukin-1β (IL-1β) mediates disc degeneration by inducing apoptotic death of nucleus pulposus (NP) cells and degradation of the NP extracellular matrix. Here, we confirmed that extracellular secretion of IL-1β via secretory autophagy contributes to disc degeneration, and demonstrate that a thermosensitive reactive oxygen species (ROS)-responsive hydrogel loaded with a synthetic growth hormone-releasing hormone analog (MR409) can protect against needle puncture-induced disc degeneration in rats.

**Methods:** The expression levels of proteins related to secretory autophagy such as tripartite motif-containing 16 (TRIM16) and microtubule-associated protein light chain 3B (LC3B) were examined in human and rat disc tissues by histology and immunofluorescence. The effects of TRIM16 expression level on IL-1β secretion were examined in THP-1 cells transfected with TRIM16 plasmid or siRNA using ELISA, immunofluorescence, and immunoblotting. The *in vitro* effects of MR409 on IL-1β were examined in THP-1 cells and primary rat NP cells using ELISA, immunofluorescence, immunoblotting, and qRT-PCR. Further, MR409 was subcutaneously administered to aged mice to test its efficacy against disc degeneration using immunofluorescence, X-ray, micro-CT, and histology. To achieve controllable MR409 release for intradiscal use, MR409 was encapsulated in an injectable ROS-responsive thermosensitive hydrogel. Viscosity, rheological properties, release profile, and biocompatibility were evaluated. Thereafter, therapeutic efficacy was assessed in a needle puncture-induced rat model of disc degeneration at 8 and 12 weeks post-operation using X-ray, magnetic resonance (MR) imaging, histological analysis, and immunofluorescence.

**Results:** Secretory autophagy-related proteins TRIM16 and LC3B were robustly upregulated in degenerated discs of both human and rat. Moreover, while upregulation of TRIM16 facilitated, and knockdown of TRIM16 suppressed, secretory autophagy-mediated IL-1β secretion from THP-1 cells under oxidative stress, MR409 inhibited ROS-induced secretory autophagy and IL-1β secretion by THP-1 cells as well as IL-1β-induced pro-inflammatory and pro-catabolic effects in rat NP cells. Daily subcutaneous injection of MR409 inhibited secretory autophagy and ameliorated age-related disc degeneration in mice. The newly developed ROS-responsive MR409-encapsulated hydrogel provided a reliable delivery system for controlled MR409 release, and intradiscal application effectively suppressed secretory autophagy and needle puncture-induced disc degeneration in rats.

**Conclusion:** Secretory autophagy and associated IL-1β secretion contribute to the pathogenesis of disc degeneration, and MR409 can effectively inhibit this pathway. The ROS-responsive thermosensitive hydrogel encapsulated with MR409 is a potentially efficacious treatment for disc degeneration.

## Introduction

Low back pain is a common health problem worldwide, with as high as 80% of diagnosed cases having a lifetime incidence 1. Back pain-related medical costs are enormous, and lost productivity imposes a further economic burden in society 2. Lumbar disc degeneration, the progressive structural failure of the intervertebral disc, has long been regarded as a major cause of chronic back pain 3, 4 and degenerated lumbar spine disorders. A number of etiological factors may contribute to disc degeneration, including aging, genetic inheritance, compromised nutrient supply, mechanical insults, and endplate pathologies 5-8. Despite the heterogeneous etiologies, it is widely accepted that intradiscal oxidative stress and inflammation are common pathomechanisms for the initiation and progression of disc degeneration 9.

Interleukin-1β (IL-1β) is the best studied pro-inflammatory cytokines implicated in disc degeneration 10. Infiltrating immunocytes such as macrophages can release IL-1β into the extracellular space, where it activates matrix metalloproteinases (MMPs) that can degrade collagen and proteoglycan components of the nucleus pulposus (NP) matrix, leading to disc degeneration 11. Both IL-1β and its receptor are upregulated in disc degeneration patients and animal models, and higher IL-1β expression level is strongly associated with greater histological severity 10, 12. Given the important contribution of IL-1β to disc degeneration in patients, IL-1β-related pathways may be promising therapeutic targets for clinical intervention.

IL-1β has been studied extensively studied in various diseases, but most have focused on IL-1β synthesis and downstream effects, while few have examined the sources and mechanisms of IL-1β secretion 13. As a cytosolic protein devoid of leader peptides, IL-1β is not secreted through the conventional endoplasmic reticulum and Golgi apparatus pathway 14-16. Rather, lysosome-independent secretory autophagy plays a major role in the secretion of IL-1β and other leaderless cytosolic proteins 14, 16. During this process, tripartite motif-containing 16 (TRIM16) recognizes IL-1β and promotes binding to microtubule-associated protein light chain 3B (LC3B) sequestration membranes, which transport IL-1β to the plasma membrane for secretion 17, 18. This secretory autophagy pathway contributes to a wide range of pathologies, including cancer, infection, and neurodegeneration, while blocking secretory autophagy can be effective in alleviating disease progression 19, 20. However, biological intervention targeting secretory autophagy of IL-1β in degenerated intervertebral discs has not been studied in detail.

The synthetic growth hormone-releasing hormone analog MR409 has been shown to suppress disease-associated inflammation in animal models of diabetes mellitus, myocardial infarction, lung damage, and vascular calcification by modulating immunocytes infiltration and IL-1β synthesis 21-23. In addition, MR409 is an antioxidant that downregulates reactive oxygen species (ROS) accumulation or blocks ROS signaling 24. ROS is an important pathogenic pathway in disc degeneration 25, 26 and an inducer of LC3B and TRIM16 expression required for secretory autophagy 27-30. However, neither the direct suppression of secretory autophagy by MR409 nor the efficacy of MR409 against disc degeneration has been examined.

As a peptide, MR409 is prone to degradation *in vivo*, particularly in the harsh environment of a degenerated disc. Therefore, a specifically designed carrier that protects MR409 for controlled targeted release may be required for therapeutic application. ROS-responsive biomaterials may be ideal carriers for delivering MR409 to degenerated discs. In addition to ROS-triggered release, polymers with ROS-responsive bonds can consume excessive ROS and thus reduce oxidative injury to disc NP cells 31. Theoretically, such a ROS-responsive biomaterial incorporating MR409 can modulate secretory autophagy in NP cells and suppress disc degeneration.

In this study, we examined the contributions of secretory autophagy to disc degeneration in human and rat and tested whether MR409 can inhibit secretory autophagy-based IL-1β secretion and ensuing inflammation *in vitro* and as well as in aged mice. Further, we examined whether a newly developed ROS-responsive biomaterial loaded with MR409 has therapeutic efficacy for treating experimental disc degeneration in rats.

## Materials and Methods

### Collection of human disc tissues

All experiments on human and animal tissues were approved by the First Affiliated Hospital of Zhejiang University (Ethical approval numbers 2020.333 and 2020.484, respectively). Human disc samples were obtained from surgical patients with written consent. Human degenerated disc samples were obtained from patients (7 from males and 3 from females; age range, 36-77 years) receiving spinal surgery for degenerative lumbar disc disorders, while non-degenerated disc samples were obtained from patients (4 from males and 2 from females; age range from 34 to 48 years) receiving spinal surgery for traumatic fractures (Table S1). The human disc tissues were cut into half immediately after dissection for immunostaining and storage. One half was snap-frozen in liquid nitrogen and stored at -80 °C. The other section was immediately fixed in 4% paraformaldehyde, embedded in paraffin, sectioned, and processed as described in subsequent sections, for immunofluorescence (IF) and immunohistochemistry (IHC).

### Needle puncture-induced disc degeneration in rats

Ten female Sprague-Dawley rats (3 months old, approximately 150 g) purchased from the institutional Animal Center were used to establish a disc degeneration model and appropriate controls. Briefly, rats were anesthetized intraperitoneally with 0.8% (w/v) pentobarbital sodium (10 μL/g body weight). The disc between coccyx vertebra 7 and 8 (Co7/8) was identified by radioscopy and marked on the skin for puncture. Rats were randomly divided into control (no disc puncture, n = 5) and model groups (disc puncture, n = 5). In the model group, the Co7/8 disc was percutaneously punctured using a 29G needle through the skin mark to a depth of about 2 mm as previously described 32. The needle was then rotated 360° and maintained inside the disc for 1 min before removal. Rats in both groups were fed regular food and water at room temperature for 8 weeks. Thereafter, animals were sacrificed and disc tissues collected.

### Histological evaluation of disc degeneration

Harvested rat discs were fixed in 4% buffered paraformaldehyde for 24 h, decalcified in 10% ethylenediaminetetraacetic acid (EDTA) solution for 1 month, embedded in paraffin, and cut into 7-μm sections. Safranin O staining and hematoxylin and eosin (HE) staining were performed to evaluate the degree of disc degeneration. Images of stained sections were evaluated by two independent raters who are blinded to the research protocol, and measurements were averaged for statistical analysis. Histological score was rated based on cellularity, morphology, and structure of the annulus fibrosus and NP regions 33, with a score of 5 indicating normal disc, 6-11 as moderately degenerated disc, and 12-15 as severely degenerated disc (Table S2).

### Immunofluorescence staining

Human and rat discs were deparaffinized, dehydrated in gradient ethanol, treated with sodium citrate buffer solution (pH 6.0) overnight at 65 °C for antigen retrieval, permeabilized with 0.5% Triton X-100 for 20 min and blocked by incubation in 5% (w/v) bovine serum albumin (BSA) for 1 h at 37 °C. Sections were then incubated overnight at 4 °C with primary antibodies: TRIM16 (1:50; Santa Cruz, sc-398851), Aggrecan (ACAN) (1:50; Proteintech, 13880-1-AP), LC3B (1:50; CST #3868), MMP13 (1:50; abcam, 39012) and NBR1 (1:50, Santa Cruz, sc-130380), followed by incubation with secondary antibodies conjugated to Alexa Fluor®488 or Alexa Fluor®555 (1:200) for 1 h at 37 °C. Sections were counterstained with 4′,6-diamidino-2-phenylindole (DAPI) for 15 min. Stained sections were examined under a fluorescence microscope (Olympus, IX83-FV3000, Tokyo, Japan) and analyzed quantitatively using ImageJ (V1.8.0, NIH, Bethesda, MD). The proportions of positively stained or co-localization cells in each specimen were counted on at least three sections of the NP region and measurements were averaged for analysis. Approximately 30 immunopositive or co-localization cells were counted on each section.

### Human THP-1 cell differentiation, transfection, oxidant treatment, and immunostaining

Human THP-1 cells were purchased from the Cell Bank of the Chinese Academy of Sciences (Shanghai, China). THP-1 cells were differentiated into macrophage-like cells by culture for 24 h in 100 nM phorbol 12-myristate 13-acetate (PMA). Differentiated cells were treated with 400 μM tert-butyl hydroperoxide (TBHP) for 2 h to induce oxidative stress in the absence or presence of 10 nM MR409. The supernatant was collected to measure IL-1β with ELISA and the cells were harvested for Western blotting or processed for immunofluorescence. In other experiments, TRIM16 expression level was first downregulated using a targeted siRNA (Genepharma, Shanghai, China). All siRNA sequences were presented in Table S3, and TRIM16#2 siRNA was used for all reported experiments. In brief, THP-1 cells were differentiated with 100 nM PMA for 24 h and transfected with 20 pM targeted siRNA or control siRNA using Lipofectamine 2000 (Invitrogen, Carlsbad, CA, USA). Alternatively, TRIM16 was overexpressed in differentiated THP-1 cells by transfection with 1 μg TRIM16-plasmid (Vigene, Shangdong, China) using Lipofectamine 2000 according to the manufacturer's instructions. The efficiency of TRIM16 plasmid transfection using lipofectamine 2000 was as high as about 80%. In separate experiments, Atg5 expression was downregulated using a targeted siRNA (Table S6).

Cells treated as described were then processed for IF by fixation in 4% paraformaldehyde for 30 min at room temperature, permeabilized with 0.5% Triton X-100 for 20 min, blocking in 5% (w/v) BSA for 1 h at 37 °C, and incubation in the indicated primary and second antibodies. Finally, a total of 30 THP-1 cells were imaged under a fluorescence microscope to determine the number, intensity, and overlap profile of LC3 and TRIM16 immunofluorescent puncta using ImageJ. Only TRIM16 and LC3 puncta of 0.1-1.0 μm diameter were counted 34, 35, 36.

### Western blotting

Cells and disc tissues treated as indicated from last section were lysed in RIPA buffer with protease inhibitor. Total cellular proteins were separated on SDS-PAGE gels and transferred to PVDF membranes (Bio-Rad). Membranes were blocked with 5% (w/v) non-fat milk for 1 h at room temperature and then incubated overnight at 4 °C with primary antibodies against one or more of the following proteins: TRIM16 (1:500, Santa Cruz, sc-398851), LC3B (1:1000, CST, #3868), ACAN (1:1000, Proteintech, 13880-1-AP), MMP13 (1:3000, abcam, 39012), SOX9 (1:1000, abcam, ab185966), NBR1 (1:400, Santa Cruz, sc-130380), GAPDH (1:5000, CST, #5174S), and (or) actin (1:5000, MultiSciences, 70-ab008-040). Anti-GAPDH was used as the gel loading control 37. The membranes were then washed in Tris-buffered saline plus Tween and incubated with HRP-conjugated secondary antibodies (1:5000). Protein bands were visualized using ECL reagent (Beyotime Institute of Biotechnology) and semi-quantified using ImageJ. Target protein expression levels (band intensities) were expressed relative to GAPDH expression.

### Rat NP cell isolation and treatment

Rats were sacrificed using an overdose of sodium pentobarbital and NP tissues were carefully collected from the caudal discs and digested with 0.2% (w/v) type II collagenase for 4 h at 37 °C. The isolated cells in supernatant were platted in DMEM/F12 medium supplemented with 10% (v/v) fetal bovine serum (FBS, Gibco, Gaithersburg, MD, USA) and antibiotics, and maintained in an incubator at 37 °C under a 5% CO_2_ atmosphere_._ The medium was changed every other day. Cells were then treated for 24 h with 10 ng/ml IL-1β, 10 nM MR409, or IL-1β either alone or in combination with MR409. Untreated cells served as controls.

### Cell viability analysis

The cytotoxicity of MR409 on NP cells was evaluated using the Cell Counting Kit-8 (CCK8, Beyotime, China) according to the manufacturer's instructions. NP cells were treated with MR409 at different concentrations (0, 0.5, 1, 2, 5, 10, 20, 30 nM) for 24 h, washed with PBS, and incubated in DMEM/F12 with CCK8 staining solution for 2 h at 37 °C. Thereafter, the optical density (proportional to viable cell number) was measured at 450 nm using a microplate reader.

### RNA isolation and real-time PCR

Total RNA was extracted from rat NP cells using TRIzol reagent (Invitrogen, USA), reverse transcribed to cDNA using a Prime Script-RT reagent kit (Sangon, Shanghai, China), and amplified using SYBR Premix Ex Taq (Sangon) and the primers presented in Table S4. The expression levels of target mRNAs were measured as described previously using GAPDH as the internal control 37. The Ct value of the target gene was standardized to that of GAPDH and relative expression calculated according to 2^-ΔΔCt^. In figures, target gene expression was normalized to that of untreated control cells.

### Age-related mice model of disc degeneration and MR409 administration

Ten 15-month-old mice were divided equally into a control group receiving daily subcutaneous injection of 100 μL phosphate-buffered saline (PBS) and a MR409 group receiving daily subcutaneous injection of 100 μL MR409 (0.1 μg/μL). After 8 months, mice were sacrificed and discs were collected.

### Radiographic analysis of disc height index

X-ray was taken for rat tails and radiographs were measured to acquire disc height using ImageJ. The disc height index (DHI) was defined as average of anterior, middle, and posterior disc heights normalized to the average of the corresponding adjacent vertebral body heights. Thus, a lower DHI value indicates more severe disc degeneration 38.

### Micro-computerized tomography (μCT)

Prior to histological analysis, paraformaldehyde-fixed samples were scanned using a μCT (μCT-100, SCANCO Medical AG, Switzerland) at 70 kV, 200 µA, 20 µm resolution, and 300 ms exposure time. Images were analyzed using *Evaluation* (V6.5-3, SCANCO Medical AG, Switzerland).

### Immunohistochemistry

Paraffin-embedded histological sections were deparaffinized in xylene, rehydrated in graded ethanol, washed with distilled water, heated overnight in 65 °C sodium citrate buffer solution (pH 6.0) for antigen recovery, washed in PBS, incubated in 0.3% (v/v) hydrogen peroxide for 20 min to block endogenous peroxidase activity, and blocked by incubation for 1 h in 5% (w/v) BSA. Sections were then incubated overnight at 4 °C with primary antibodies against ACAN (1:100; Proteintech, 13880-1-AP) and MMP13 (1:150; abcam, 39012). The next day, sections were washed in PBS containing Tween and incubated for 1 h at room temperature with horseradish peroxidase (HRP)-labeled secondary antibodies. Immunolabeling was visualized using diaminobenzidine (DAB). The proportion of positively stained cells to total cells was calculated from three fields of the NP region. In all treatment groups, approximately 30 immunopositive cells were counted.

### Terminal deoxynucleotidyl transferase dUTP nick end labeling (TUNEL) of apoptotic cells

Apoptotic cells were counted using a commercial TUNEL assay (*In situ* Cell Death Detection Kit, Roche, Mannheim, Germany) according to the manufacturer's instructions. Briefly, after deparaffinization and dehydration in gradient ethanol, sections were permeabilized with 0.5% Triton X-100 for 10 min at room temperature, incubated with TUNEL mixture for 60 min at 37 °C, and counterstained with DAPI for 10 min. Sections were imaged under a fluorescence microscope (Nikon Eclipse Ti-SR, Japan) to determine the proportion of apoptotic cells to total cells.

### Preparation and characterization of MR409-loaded vesicles

The ROS-responsive block polymer methoxy poly(ethylene glycol)-b-poly(propylene sulfide) (mPEG_20_-b-PPS_30_) was prepared according to a previously reported method (see supplementary information) 39. MR409 was then loaded into mPEG_20_-b-PPS_30_ (PPS-PEG)-based vesicles via a solvent exchange method. Briefly, amphiphilic PPS-PEG (5 mg) block copolymer was dissolved in 1 mL tetrahydrofuran (THF), followed by slow drop-wise addition to 2 mL of 0.1 μg/μL MR409 aqueous solution. The organic solvent was removed using a rotavapor and MR409-loaded vesicles (vesicle/MR409) purified by three centrifugations at 4000×g (10 min/each) and washing with PBS using an Amicon Ultra-15 centrifugal filter unit (Merck-Millipore, USA). Transmission electron microscopy (TEM, HT7700, Hitachi, Japan) was used to examine vesicle morphology, while dynamic light scattering (DLS, Zetasizer 3000, Malvern, Southborough, MA) was used to measure the hydrodynamic diameter of vesicles at room temperature.

A labeled MR409 analog with one lysine replaced with a fluorescein isothiocyanate (FITC)-label lysine (Sangon, Shanghai, China) was used to quantify vehicle loading and release of MR409. After vesicle/MR409 preparation and purification, the supernatant was collected and UV absorption measured to calculate the amount of MR409 analog released according to a standard curve. The vesicle/MR409 aqueous suspension was then stored at -20 °C until use.

### Preparation and characterization of MR409-loaded vesicles in an injectable hydrogel

A thermosensitive triblock poly(lactic-co-glycolic acid)-b-poly(ethylene glycol)-b-poly(lactic-co-glycolic acid) copolymer (PLGA-PEG-PLGA) with a molecular weight (in Da) ratio of ~200:5000:200 (LA:GA = 65:35) was purchased from Xi'an Ruixi Biological Technology (China). A 10% (w/v) PLGA-PEG-PLGA solution containing vesicle/MR409 (6.3/1.0 mg/mL) demonstrated a solution-to-gel transition at 34 °C under physiological conditions.

The rheological behaviors of the thermosensitive hydrogel were tested using a RS6000 stress-controlled rheometer (HAAKE, Germany). Briefly, PLGA-PEG-PLGA was dissolved in 0.9% NaCl aqueous solution (10% w/v) at 4 °C. A 1 mL sample was added to the gap (0.3 mm) between a small flat plate (diameter 60 mm) and a larger basal plate. The temperature increase rate was set to 1 °C per min and angular frequency to 10 rad/s at 1% strain.

The release profile of fluorescein-labeled MR409 analog from the composite hydrogel was determined with or without additional H_2_O_2_ (100 μM). A 1-mL sample of hydrogel containing vesicle/MR409 analog was sealed in a dialysis cassette (cutoff MW 50 kDa) and immersed in a beaker containing 50 ml PBS or PBS/H_2_O_2_ at 37 °C. The concentrations of released MR409 analog were quantified at each time point by UV absorption.

### *In vitro* assessment of hydrogel biocompatibility with rat NP cells

The hydrogel solution was placed on ice to prevent gel formation. Next, 1 × 10^6^/mL NP cells were resuspended in the hydrogel solution and plated in regular culture media at 37 °C under a 5% CO_2_ atmosphere. Cell seeding was verified by microscopy. A live/dead cell viability assay was performed on days 1, 3, and 5 after plating in which live cells were distinguished by calcein staining and dead cells by propidium iodide staining. The viability was calculated by the ratio of live cells/total cells and representative images obtained with a Nikon A1R confocal laser scanning microscope (Nikon, Tokyo, Japan).

### Treating puncture-induced disc degeneration with MR409-loaded hydrogel

The protective efficacy of this MR409-loaded hydrogel was then tested against puncture induced disc degeneration. Fifty female rats were equally divided into 5 groups: a control group (no disc puncture and no injection), PBS group (PBS injection following disc puncture), MR409 group (MR409 injection following disc puncture), hydrogel group (hydrogel injection following disc puncture), and hydrogel+MR409 group (MR409-encapsulated hydrogel injection following disc puncture). Except for animals in the control group, the Co7/8 disc was percutaneously punctured as described, followed by a 3-μL injection of the indicated substance. At postoperative weeks 8 and 12, 5 rats in each group were sacrificed and the tails were dissected for imaging and histology.

### Magnetic resonance (MR) imaging and assessment

The rat tails were imaged using a 3.0T Intera Achieva 3.0 MR scanner (Philips) to acquire T2-weighted MR images, which is the most common MR sequence used for clinical assessment of the intervertebral disc 40. Imaging protocol: spin echo repetition time, 2700 ms; echo time, 99 ms; number of excitations, 8; field of view, 5 cm; slice thickness, 1.5 mm. A surgeon who is blinded to animal treatment history assessed the degree of disc degeneration using the Pfirrmann scale (Table S5), which is based on structure, signal intensity distribution, and height of the intervertebral disc (with grade I indicating least degeneration and grade V most severe degeneration) 40.

### Statistical analysis

Each experiment was performed at least three times. Data were expressed as mean + standard deviation (SD). The normality of the data was tested before statistical test. Student's *t*-test was used to compare two groups, and one-way analysis of variance (ANOVA) with a post-hoc Tukey tests were performed among more groups. Data were analyzed and graphed using GraphPad Prism (Version 5.0, GraphPad Software, San Diego, CA, USA). Statistical significance was set at *p* < 0.05.

## Results

### Secretory autophagy was elevated during disc degeneration

**Figure 1:**
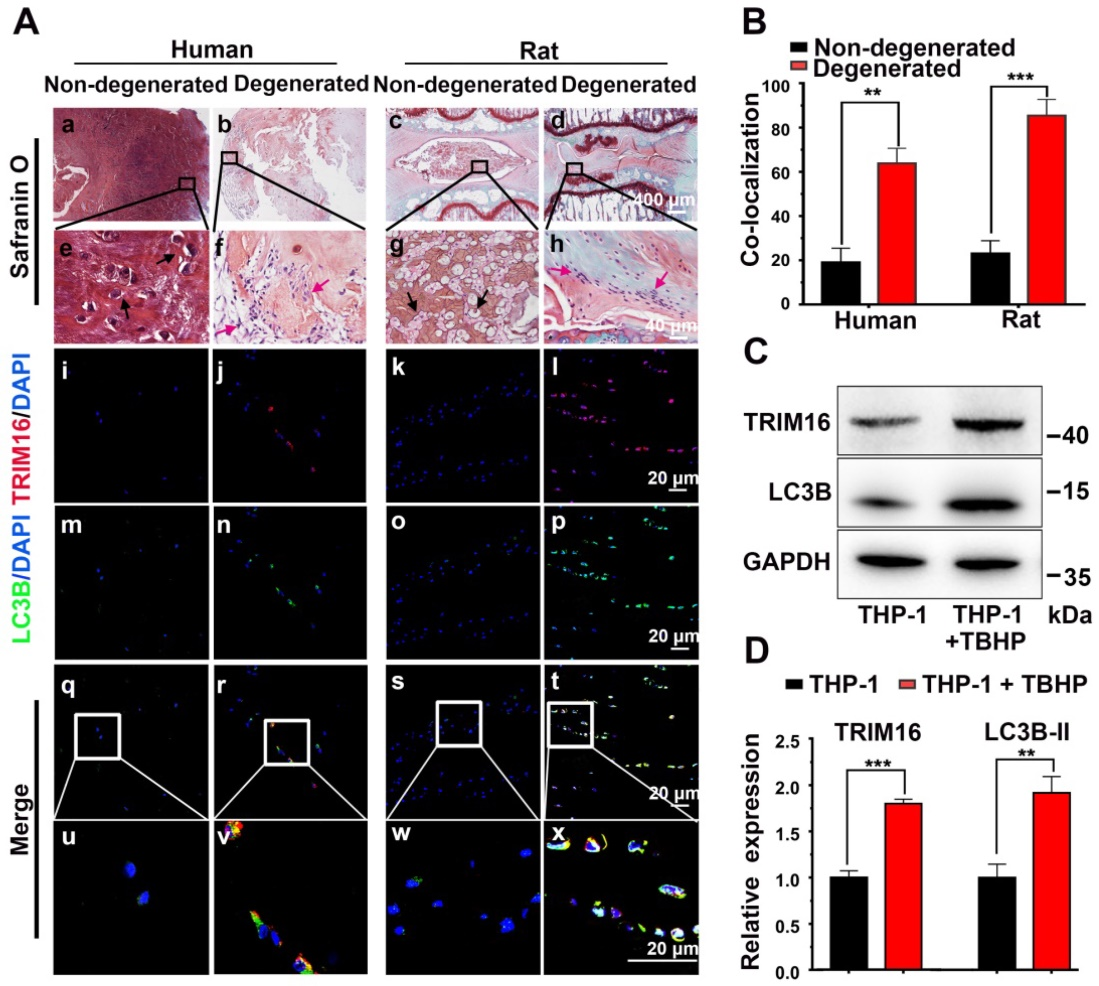
** Activation of secretory autophagy in degenerated human and rat discs. (A, a-h)** Safranin-O-stained sections showing reduced proteoglycan-rich matrix (red), fewer chondrocyte-like cells (black arrows), and more numerous infiltrating inflammation cell clusters (red arrows) in degenerated human and rat discs compared to non-degenerated discs. **(A(i-x), B)** Immunofluorescence images demonstrating significantly greater expression of the secretory autophagy-related proteins TRIM16 (red) and LC3B (green) as well as greater TRIM16-LC3B co-localization (merged yellow) in human and rat degenerated discs. **(C and D)** Immunoblot showing higher LC3B and TRIM16 proteins levels in human (macrophage-like) THP-1 cells under oxidative stress (TBHP, 400 µM, 2 h) (n = 3). Data are expressed as the mean + SD. Group means were compared with independent samples *t*-test. **p < 0.01; ***p < 0.001.

Based on previous observations that IL-1β elevated in degenerated discs 10 and IL-1β was mainly secreted through the secretory autophagy pathway in other pathogenic processes 14, 16, we examined whether secretory autophagy was involved in disc degeneration. Safranin O staining revealed that degenerated discs from both human and rat puncture model exhibited a reduced proteoglycan-rich matrix, fewer chondrocyte-like cells, and more metatypical clustered (infiltrating) cells compared to corresponding non-degenerated discs (Figure 1A(a-h)). Further immunofluorescence staining revealed enhanced expression of the secretory autophagy cargo receptor TRIM16 and induction of autophagy (represented by increased fluorescence of LC3B and reduced fluorescence of NBR1) were enhanced in degenerated discs (Figure 1A(e-t) and S2E). The proportion of TRIM16 and LC3B co-localization cells (the percentage of cells with both markers) reached approximately 60% in human degenerated discs and 85% in rat degenerated discs (Figure 1B), both significantly higher than corresponding non-degenerated discs (~20%). As TRIM16 and LC3B are important biomarkers for secretory autophagy, this high level of co-localization strongly suggested that upregulated secretory autophagy contributed to the pathogenesis of disc degeneration.

Further immunofluorescence staining for CD68 and TRIM16 indicated that most TRIM16-labeled cells in the degenerated discs were infiltrating macrophages rather than resident NP cells (Figure S3). Therefore, human monocytic THP-1 cells were utilized as a model to examine the activation status of secretary autophagy *in vitro*. As oxidative stress is a major mediator of disc degeneration, we exposed (M0 differentiated) THP-1 cells to TBHP (an exogenous ROS donor) and examined TRIM16 and LC3B expression. Consistent with enhanced secretory autophagy* in vivo*, TBHP markedly upregulated the expression levels of both TRIM16 and LC3B in differentiated THP-1 cells (Figure 1C-D). Collectively, these results suggested activation of secretory autophagy in degenerated discs mediated by macrophages in response to ROS accumulation and ensuing oxidative stress.

### TRIM16 mediated IL-1β secretion under oxidative stress

**Figure 2:**
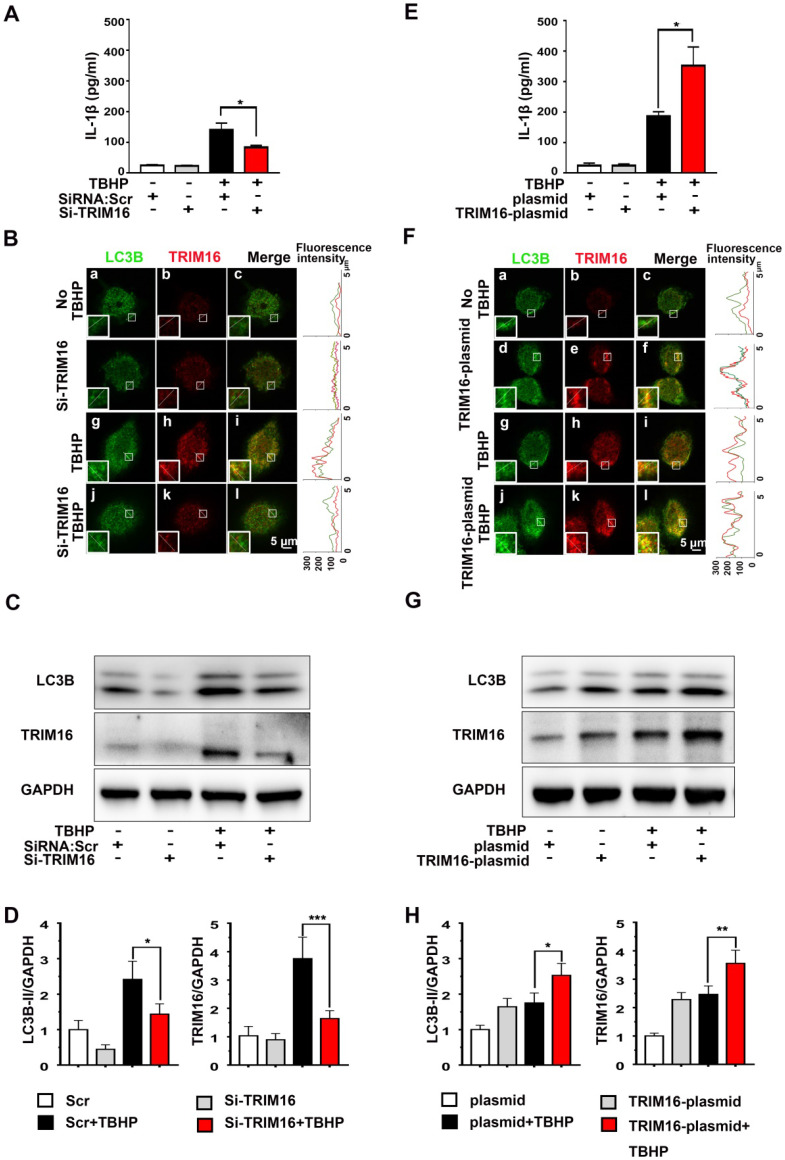
** Oxidative stress induced TRIM16-mediated, secretory autophagy-dependent IL-1β secretion from THP-1 cells.** THP-1 cells were transfected with a TRIM16 overexpression plasmid or siRNA, and then treated with 400 µM TBHP for 2 h. **(A)** TRIM16 knockdown suppressed IL-1β secretion under oxidative stress as measured by ELISA. **(B)** Immunofluorescence demonstrated that knockdown also decreased LC3B (green) and TRIM16 (red) co-localization (merged yellow) in THP-1 cells under oxidative stress. Magnification showed the representative co-localization of LC3B and TRIM16. **(C and D)** TRIM16 knockdown and concomitant LC3B down-regulation under oxidative stress were confirmed in immunoblotting. **(E-H)** Conversely, TRIM16 overexpression enhanced IL-1β secretion **(E)** and expression of LC3B under oxidative stress **(F-H).** Data are expressed as the mean + SD. Group means were compared by independent samples t-test, n = 3. *p < 0.05; **p < 0.01; ***p < 0.001.

To examine if enhanced secretory autophagy actually results in greater intradiscal IL-1β secretion, we measured IL-1β release from oxidant-stimulated human differentiated THP-1 cells transfected with TRIM16-plasmid or TRIM16 siRNA (Figure S4). Under oxidative stress, IL-1β secretion was significantly attenuated by TRIM16 siRNA-mediated knockdown compared to control cells transfected with empty vector (Figure 2A). Moreover, immunofluorescent staining further demonstrated that TRIM16 knockdown reduced the expression of LC3B and TRIM16 and their interaction in the cell, as reflected by decreased co-localization of LC3B and TRIM16 (Figure 2B). The reduced LC3B and TRIM16 protein expression levels were further confirmed by immunoblotting (Figure 2C and 2D). Alternatively, TRIM16 overexpression with the plasmid enhanced TBHP-induced upregulation and co-localization of TRIM16 and LC3B, as well as secretory autophagy-based IL-1β secretion (Figure 2E-H). In addition, TRIM16 overexpression and knockdown did not influence IL-1β secretion in the absence of TBHP. These findings suggested that in degenerated discs IL-1β was secreted from intradiscal macrophages via secretory autophagy in response to oxidative stress.

### MR409 reduced IL-1β secretion from THP-1 cells

**Figure 3:**
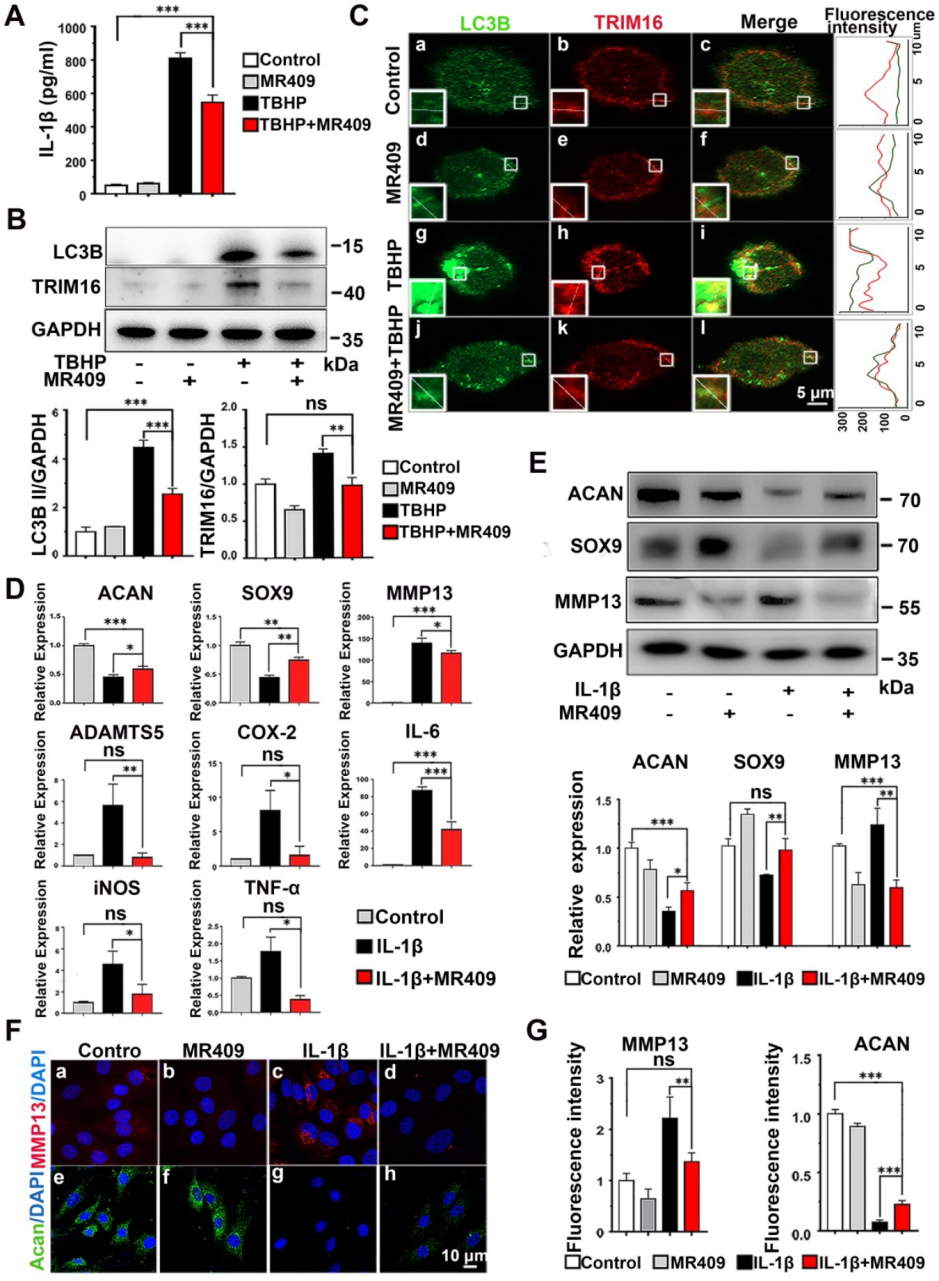
** MR409 attenuated secretory autophagy-mediated IL-1β secretion from THP-1 cells and suppressed the activation of IL-1β-induced pro-inflammatory pathway and matrix catabolism in disc nucleus pulposus cells. (A)** MR409 significantly inhibited tert-butyl hydroperoxide (TBHP)-induced IL-1β secretion into the supernatant as measured by ELISA. **(B)** MR409 suppressed TRIM16 and LC3B protein expression levels in THP-1 cells with immunoblotting. GAPDH expression was used as the gel loading control. **(C)** MR409 attenuated TBHP-induced expression and co-localization of LC3B in THP-1 cells as evidenced by immunofluorescence staining. Magnification showed the representative co-localization of LC3B and TRIM16. **(D)** IL-1β-induced downregulation of anabolic factor genes (ACAN and SOX9) and upregulation of both catabolic factor genes (MMP13 and ADATMS5) and inflammatory mediator genes (COX-2, IL-6, iNOS and TNF-α) in rat NP cells were effectively reversed by MR409. mRNA expression levels were measured with qRT-PCR. **(E)** MR409 reversed IL-1β-mediated induction of ACAN, MMP13, and SOX9 protein expression in rat NP cells. **(F and G)** Immunofluorescence images showed that MR409 abrogated the IL-1β-induced decrease in ACAN expression (green) and increase in MMP13 expression (red) in rat NP cells. Data are expressed as the mean + SD of three independent cultures. Means were compared by one-way ANOVA with post-hoc Tukey tests. *p < 0.05; **p < 0.01; ***p < 0.001.

Activation of secretory autophagy in degenerated discs (Figure 1) and induction of IL-1β release under oxidative stress (Figure 2) suggested that ROS and TRIM16/LC3B reducer, such as MR409, may be effective against disc degeneration. As a first step in evaluating the therapeutic efficacy of MR409, we examined its effects on ROS-induced expression of proteins associated with secretory autophagy and on IL-1β secretion from THP-1 cells (Figure 3A and Figure S5). Indeed, MR409 significantly suppressed THBP-induced secretion of IL-1β (Figure 3A), and inhibited TRIM16 and LC3B expression (Figure 3B) as well as TRIM16-LC3B co-localization (Figure 3C). Together, findings suggested that MR409 could attenuate oxidative stress-induced secretory autophagy and consequent IL-1β secretion from macrophages.

### MR409 suppressed IL-1β-induced matrix catabolism in NP cells

In addition, we examined the effects of MR409 on NP cells in culture. MR409 significantly increased the number of NP cells at 10 nM (Figure S6). As such, this concentration was applied for subsequent studies. It is well known that IL-1β promotes expression of MMPs and suppresses ECM synthesis in NP cells, leading to disc degeneration 21, 41. Therefore, the effects of MR409 on both matrix construction and catabolism were examined 42-44. As shown in Figure 3D, IL-1β treatment downregulated mRNA expression levels of the anabolic factors ACAN and SOX9, and upregulated mRNA expression of the catabolic factors MMP13 and ADATMS5 as well as the inflammation mediators IL-6, iNOS, COX-2, and TNF-α, indicating that IL-1β acted to suppress matrix generation in NP cells and promoted inflammatory signaling. In addition, the anabolism-related proteins ACAN and SOX9 were downregulated at the protein level while the catabolism-related protein MMP13 was upregulated following IL-1β stimulation (Figure 3E). These changes in protein expression were further confirmed by immunofluorescence (Figure 3F and 3G). All of these effects were effectively reversed by MR409 co-treatment, suggesting that MR409 can prevent IL-1β-induced pro-inflammatory signaling and matrix catabolism in NP cells.

### Subcutaneous injection of MR409 delayed age-related disc degeneration in mice

We then examined if these protective effects of MR409 could suppress age-related disc degeneration *in vivo.* In mice, spontaneous disc degeneration emerges at approximately 14 months of age and proceeds progressively thereafter 45, so we administered MR409 (experimental group) or saline (control group) daily for 8 months starting at 15 months by subcutaneous injection Thereafter, radiographic evaluations and histological analyses were performed to assess the degree of disc degeneration.

**Figure 4:**
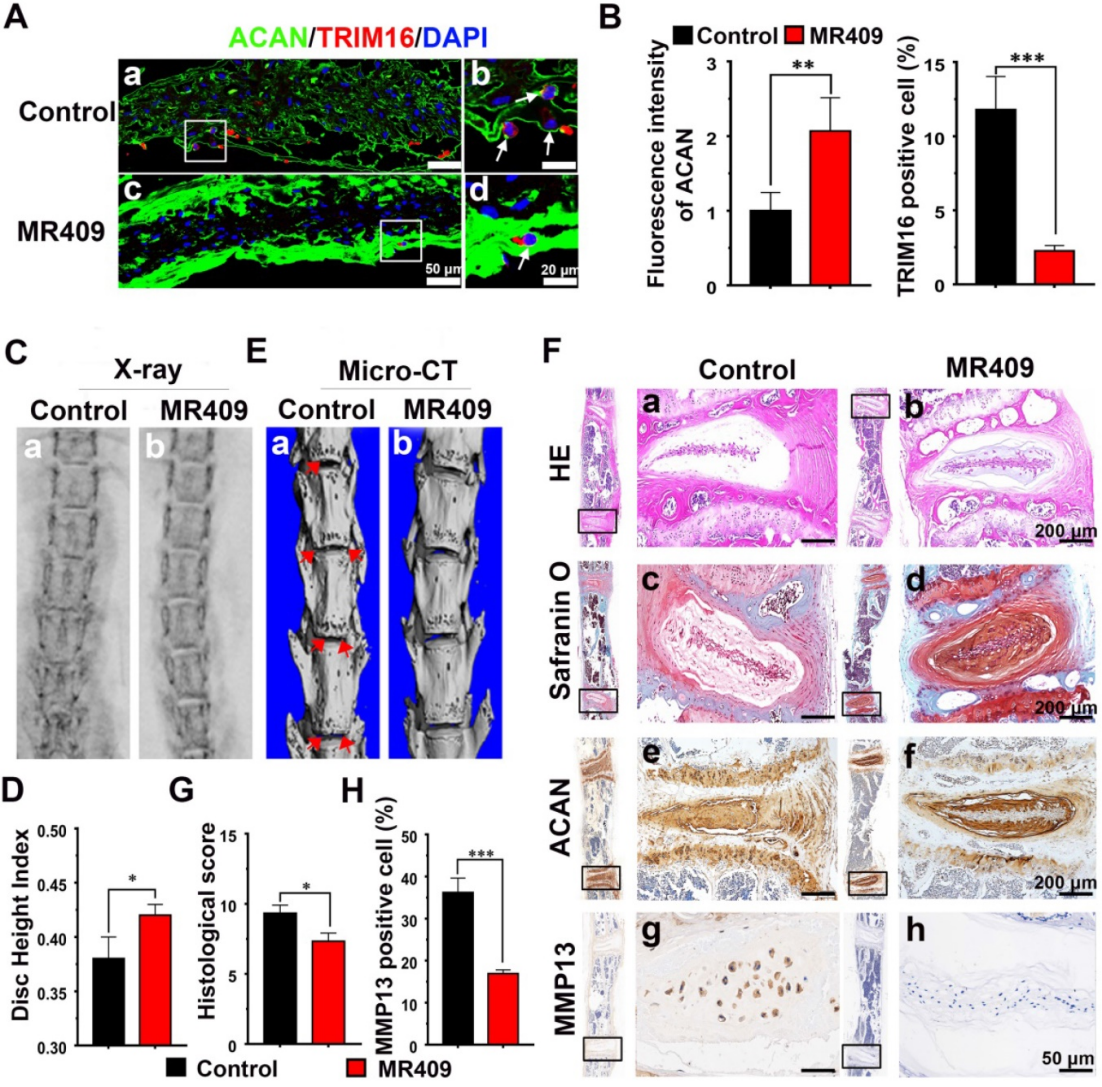
** Subcutaneous administration of MR409 delayed age-related disc degeneration in mice through inhibiting secretory autophagy. (A and B)** Representative immunofluorescence images of the nucleus pulposus (NP) region **(A)** and analysis **(B)** of TRIM16 (red) and ACAN (green) immunoexpression in lumbar disc sections from 23-month-old mice receiving daily subcutaneous injection of saline (control group) or MR409 for 8 months. MR409 inhibited secretory autophagy as evidenced by TRIM16 downregulation and promoted expression of matrix proteoglycan ACAN. **(C and D)** Representative X-ray images (C) and disc height index (DHI) measurements (D) of control and MR409 group mice demonstrated that MR409 treatment alleviated disc height loss. **(E)** Representative micro-CT images of the lumbar spine revealed that administration of MR409 decreased osteophyte formation. **(F)** Representative images of HE-stained, Safranin-O-stained, and ACAN/MMP13-immunostained disc sections from control and MR409 mice. **(G and H)** Histological evaluation of Safranin O-stained sections based on cellularity, morphology, and structure of the annulus fibrosus and NP (G) and quantitation of MMP13-positive cells (H) demonstrated that MR409 treatment protected against age-related disc degeneration in mice. Data are expressed as the mean + SD of five mice per treatment group. Means were compared with independent samples *t*-tests. *p < 0.05; **p < 0.01; ***p < 0.001.

Immunofluorescence staining of the NP region revealed that MR409 significantly upregulated matrix proteoglycan ACAN expression and reduced autophagy signaling in aged mice (Figure 4A and 4B). Moreover, MR409 treatment decreased LC3B expression and apoptosis rate of NP cells in the discs of aged mice (Figure S7). On X-ray images, the mean DHI was significantly greater in the MR409-treated group compared to the saline-injected control group (Figure 4C and 4D), suggesting that subcutaneous MR409 treatment can alleviate disc height loss. Micro-CT further revealed that the discs of MR409-treated mice had fewer osteophytes, the overgrown bone tissue regions at the vertebral margins considered a compensatory response to disc degeneration 46, 47 (Figure 4E). In addition, histology revealed more chondrocyte-like cells and proteoglycan-rich matrix in discs of the MR409 group (Figure 4F(a-d)). Also, histological degeneration score was significantly lower in the MR409 group (Figure 4G). Finally, elevated ACAN expression and reduced MMP13 expression were observed in MR409-treated discs by immunohistochemical staining (Figure 4F(e-h) and 4H). Collectively, these results indicated that subcutaneous administration of MR409 could suppress the secretory autophagy pathway and alleviate age-related disc degeneration in mice.

### Synthesis and physical characterization of a thermosensitive hydrogel containing ROS-responsive MR409-loaded vesicles

**Figure 5:**
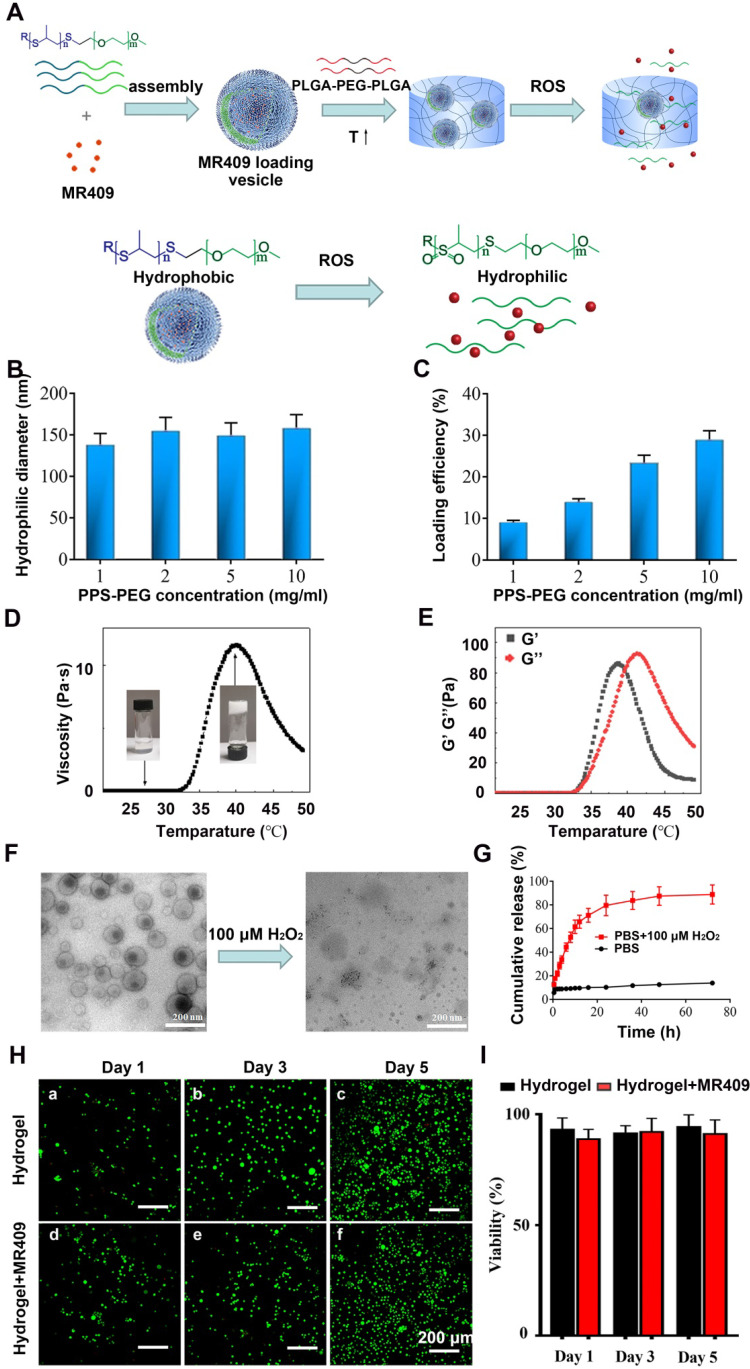
** A new injectable thermosensitive hydrogel with ROS-sensitive MR409-loaded vesicles demonstrates sustained controlled release and biocompatibility. (A)** Schematic illustration of the thermosensitive hydrogel loaded with ROS-responsive PPS-PEG vesicles for controlled release of MR409. **(B)** Hydrodynamic diameters and **(C)** MR409-loading efficiencies of PPS-PEG vesicles. **(D)** Viscosity of the PLGA-PEG-PLGA solution containing MR409-loaded vesicles as a function of temperature. **(E)** G' and G'' of the PLGA-PEG-PLGA solution containing MR409-loaded vesicles as a function of temperature. **(F)** Change in the morphology of MR409-loaded vesicles in the presence of external H2O2 (100 µM). **(G)** Cumulative release of MR409 from PPS-PEG vesicles or hydrogel containing vesicles in the presence of 100 µM H_2_O_2_. **(H and I)** Images of live/dead cell staining (H) and quantitation (I) of rat NP cells cultured with hydrogel confirming good biocompatibility. Data are expressed as the mean + SD.

Reduced pH and elevated activities of matrix catabolic enzymes in the discs not only directly lead to NP degeneration but can also damage and impair the activities of potential reparative proteins such a MR409. Therefore, a nanocarrier conferring protection and controlled release may enhance the therapeutic efficacy of MR409. Also, an injectable hydrogel is advantageous for targeted *in situ* application. Since degenerated discs also exhibit ROS accumulation 48, 49, water soluble MR409 was loaded into ROS-responsive vesicles composed of a PPS-PEG amphiphilic polymer, and these loaded vesicles were then embedded in a thermosensitive PLGA-PEG-PLGA hydrogel for protection and controlled release 39 (Figure 5A).

The obtained spherical vesicles averaged approximately 150 nm in diameter (Figure 5B) as measured by DLS and were efficiently loaded with MR409, especially at higher polymer concentrations (Figure 5C). Vesicles containing 0.8 mg MR409 per 5 mg PPS-PEG vesicles were used for both physical characterization and therapeutic efficacy experiments.

The sol-gel transition and thermosensitive rheological properties of the PLGA-PEG-PLGA solution were demonstrated in Figures 5D and 5E. The relatively low viscosity and modulus of PLGA-PEG-PLGA solution below 30 °C indicated a liquid state, which supports injectability at room temperature. As temperature increased, the viscosity and modulus of the PLGA-PEG-PLGA solution increased rapidly, resulting in gel formation driven by hydrophobic association 50. The hydrogel attained a stable solid state around 37 °C with viscosity > 10 Pa·s and modulus of nearly 100 Pa. As temperature increasing further over 42 °C, the G' decreased below G'', indicating transition to a liquid state due to deterioration of the PLGA-PEG-PLGA micellar structure 51.

It was also critical to examine these properties under pathological H_2_O_2_ concentrations as present in degenerated discs 52. Under this condition, the vesicle structure disappeared (Figure 5F), confirming the ROS-responsive nature of the PPS-PEG vesicles. As a result, MR409 release was rather slow in PBS but substantially accelerated after addition of H_2_O_2_ (Figure 5G). The encapsulation of vesicle/MR409 into the hydrogel did not impede this ROS-responsive release of MR409, which may be attributed to the high permeability of hydrogel for small molecules.

The influence of the composite hydrogel on cell viability was also investigated using a live/dead cell staining assay. With longer culture time, greater numbers of viable cells were observed on both hydrogel and composite hydrogel without significant difference at any time point, indicating good biocompatibility (Figure 5H and 5I). These observations suggested that the injectable hydrogel containing ROS-responsive vesicles was a reliable delivery system for controlled release of MR409.

### ROS-responsive MR409-loaded vehicles in hydrogel alleviated puncture-induced disc degeneration in rats

**Figure 6:**
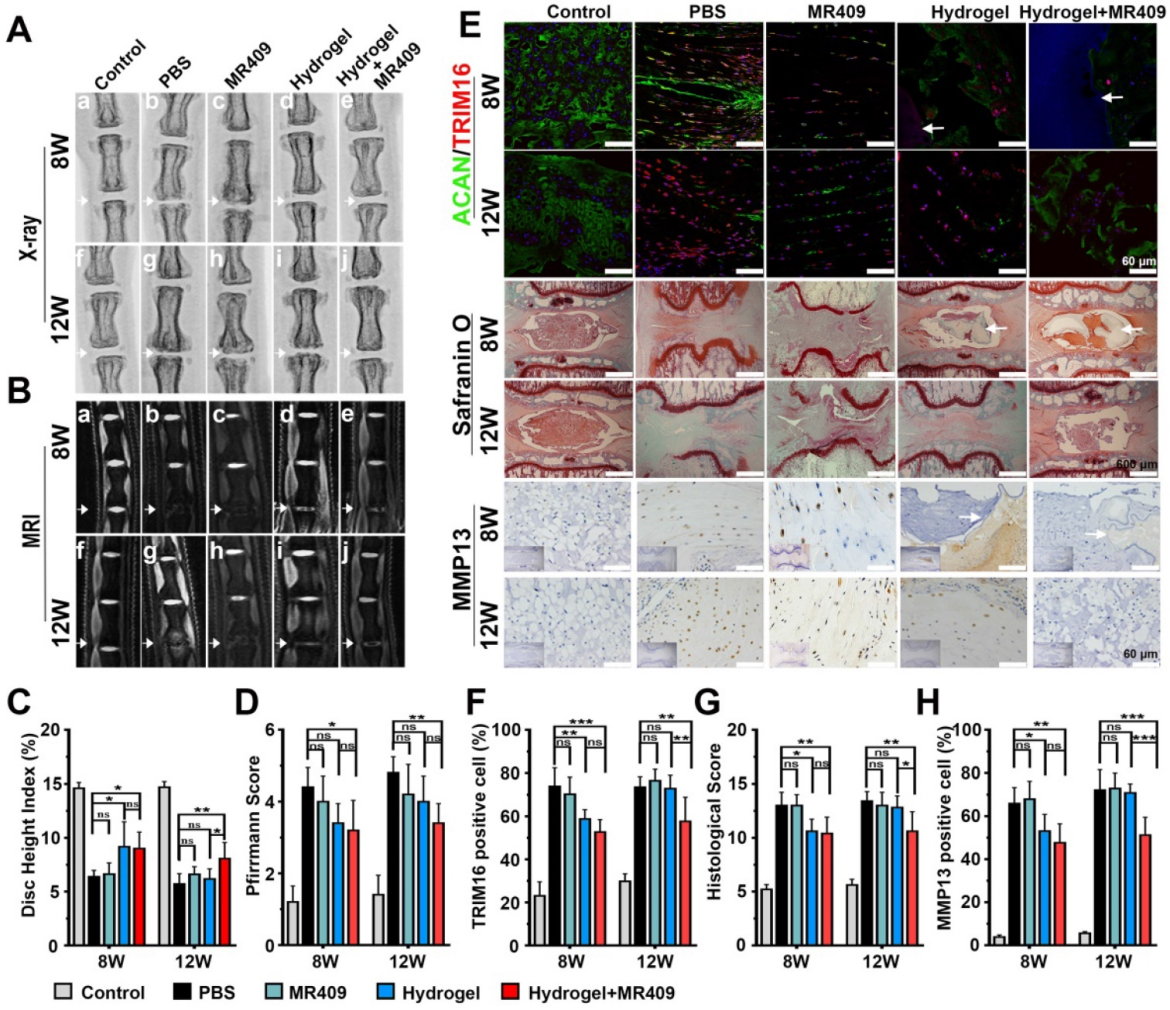
** The thermosensitive hydrogel with ROS-responsive MR409-loaded vesicles attenuated needle puncture-induced disc degeneration in rats. (A and B)** Representative spine X-ray (A) and MR images (B) from the 5 experimental groups at 8 and 12 weeks. **(C and D)** Disc height index measured on X-ray images (C) and Pfirrmann score assessed on MR images (D) showed that the MR409-encapsulated hydrogel significantly alleviated disc height loss and attenuated disc degeneration at both 8 and 12 weeks after disc puncture. **(E)** Representative immunofluorescence staining of ACAN (green) and TRIM16 (red), Safranin O staining, and immunohistochemical staining of MMP13 in experimental discs at postoperative weeks 8 and 12. Arrows indicate residual hydrogel. **(F)** Reduced numbers of TRIM16-positive cells in discs, indicating that MR409-encapsulated hydrogel inhibited secretory autophagy. **(G and H)** Histological score (G) and quantitation of MMP13-positive cells (H) showed that MR409-encapsulated hydrogel alleviated disc degeneration at both 8 and 12 weeks after puncture surgery. Data are expressed as the mean + SD of 5 rats per treatment group. Group means were compared by one-way ANOVA with post-hoc Tukey tests. ns, not significant; *p < 0.05; **p < 0.01; ***p < 0.001.

Finally, this newly developed injectable thermosensitive hydrogel was tested for alleviating disc degeneration in rat model 32. Rats were injected with PBS, MR409, hydrogel, or hydrogel containing vesicle/MR409 into the disc center following needle puncture, while a control group received no needle puncture. At postoperative week 8, X-rays demonstrated significantly greater DHI values in the hydrogel and hydrogel+MR409 groups compared to the PBS- and MR409-treated groups. Further, the hydrogel+MR409 group demonstrated greater DHI than the PBS-treated group at postoperative week 12 (Figure 6A and 6C), while there was no significant difference in DHI between PBS and MR409 groups at both 8 and 12 weeks postoperatively. The hydrogel and hydrogel+MR409 groups exhibited greater signal intensity on T2-weighted MR images compared to MR409 and PBS groups at 8 weeks, suggesting a higher hydrogen oxide content in the NP. At 12 weeks, T2-weighted MR signal was apparently greater in the discs in the hydrogel+MR409 group, as compared to those in the PBS group (Figure 6B). Pfirrmann disc degeneration score based on structure, signal intensity distribution, and intervertebral disc height 40 revealed that hydrogel+MR409 reduced disc puncture-induced degeneration at both 8 and 12 weeks (Figure 6D).

Immunofluorescence analysis also showed that MR409-encapsulated hydrogel treatment suppressed TRIM16 expression and activated ACAN expression at both 8 and 12 weeks. In addition, MR409-encapsulated hydrogel treatment inhibited apoptosis and autophagy (represented with decreased LC3B expression and enhanced NBR1 expression at both 8 and 12 weeks (Figure S8 and S2). Hydrogel treatment alone inhibited secretory autophagy and promoted ACAN expression at 8 weeks but not at 12 weeks. No significant differences in secretory autophagy and ACAN expression levels were observed between PBS and MR409 groups (Figure 6E and 6F). In addition, the hydrogel remained in the disc at 8 weeks but disappeared by 12 weeks. At postoperative week 8, histological staining showed that both hydrogel+MR409 and hydrogel treatments alleviated tissue disorganization of the punctured disc, as compared to MR409 and PBS treatment groups. At 12 weeks, however, only hydrogel+MR409 treatment attenuated disc degeneration (Figure 6E and 6G), while there was no significant difference in histological disc degeneration score between MR409 and PBS treatment groups at 8 and 12 weeks. Immunohistochemical staining further demonstrated that hydrogel+MR409 treatment downregulated MMP13 expression compared to PBS treatment at both 8 and 12 weeks (Figure 6E and 6H). Collectively, these findings suggested that direct application of our ROS-responsive MR409-encapsulated hydrogel could protect punctured discs from degeneration.

## Discussion

Given the essential roles of lumbar disc degeneration in back pain and a variety of degenerative lumbar disorders, novel therapeutic strategies are urgently needed to slow or reverse the progression of disc degeneration. In the present study, we demonstrate for the first time that IL-1β released through secretory autophagy contributes to disc degeneration in both human and animal models and that pharmacologic inhibition of this pathway can ameliorate age-related and injury-induced disc degeneration in rodents. Moreover, our newly developed MR409-encapsulated ROS-responsive thermosensitive hydrogel demonstrated to some extents sustained intradiscal release and greater therapeutic efficacy against needle puncture-induced disc degeneration in rats, illustrating a potentially promising therapeutic strategy for treating disc degeneration through inhibiting secretory autophagy.

Disc degeneration and treatment have been studied for decades, but related molecular pathomechanisms are still not fully elucidated 53. It is well established, however, that extracellular IL-1β contributes to the progression of disc degeneration 54 and can even stimulate disc degeneration *in vitro* 55. Based on these findings, several studies have examined IL-1β signaling blockade for treatment of disc degeneration, with most focusing the inhibition of intracellular production or downstream effects on resident disc cells 12, 56. In our study, induction of IL-1β through secretory autophagy and more co-localization of secretory autophagy markers (TRIM16 and LC3B) in degenerated discs pointed that secretory autophagy may contribute to disc degeneration with IL-1β secretion. Therefore, we demonstrate a potential alternative strategy targeting the secretory autophagy pathway responsible for IL-1β secretion 13, 16. One advantage of this strategy is that there are several reliable biomarkers for secretory autophagy such as TRIM16 to facilitate further mechanistic investigations and monitor therapeutic response.

The primary physiological functions of autophagy are cell degradation and homeostatic maintenance under stress 57-60, but growing evidence indicates that autophagy is required for secretion of IL-1β and IL-18 19. Unlike conventional degradative autophagy, secretory autophagy does not contribute to lysosomal degradation of IL-1β but rather facilitates extracellular accumulation 17. While secretory autophagy and degradative autophagy share some common substrates and effector molecules, there are a few molecules that control pathway divergence. For example, secretory autophagy relies on TRIM16 to identify and transfer IL-1β to the plasma membrane, from which both are secreted 61. Moreover, TRIM16 knockout disrupts the distribution of IL-1β in LC3-II membranes and thus impairs IL-1β release 62, underscoring the potential of TRIM16 as a therapeutic target and a biomarker. Here, based on the ELISA results of TRIM16 overexpression and knockdown, we found that TRIM16 indeed induced IL-1β secretion under oxidative stress. Further, Western blot and immunofluorescent experiments demonstrated that TRIM16 induced the expression and subcellular localization of another secretory autophagy-related molecule, LC3B. Collectively, findings suggested that TRIM16 was a key node in the secretory autophagy pathway and thus a promising target for therapeutic intervention.

It is well established that ROS promote disc degeneration by inducing degradative autophagy and matrix degradation 29, 65. There are also reports that TRIM16 may be an oxidative stress-induced molecule, thereby linking oxidative stress to secretory autophagy 27, 28. The current work suggested that in the presence of a ROS donor, secretory autophagy-related proteins were enhanced and more IL-1β was secreted from THP-1 cells. These findings suggested that oxidative stress promoted disc degeneration at least in part by activating secretory autophagy and IL-1β secretion.

Intradiscal application of therapeutic compounds for disc repair and regeneration can increase local concentration for greater efficacy and prevent side effects 66, 67. Due to the harsh environment of the degenerated disc, however, certain compounds are prone to degradation and absorption, which may not function with only once treatment 68. Therefore, this may be why the secretory autophagy inhibitor MR409 had little protective efficacy against experimental disc degeneration when injected directly into the disc. Biomaterials, which are able to release encapsulated bioactive molecules under specific conditions, may be a practical solution 69. Given the roles of ROS in secretory autophagy and disc degeneration 70, we designed a ROS-responsive material to efficiently encapsulate, protect, and control the release of MR409, thereby facilitating its therapeutic efficacy against disc degeneration. Moreover, hydrogels have a microstructure similar to that of disc extracellular matrix 68, 71, 72 and so can partially restore the local microenvironment for resident disc cells to survive and proliferate 73. In addition, hydrogels can prevent nanoparticle suspensions from leaking and absorbing 74, 75. However, the hydrogel alone appeared to have limited efficacy against disc degeneration, which might be attributed to the fact that the hydrogel does not obviously respond to ROS. The developed composite system, an injectable thermosensitive hydrogel loaded with ROS-responsive MR409 vesicles, provided sustained release of MR409 and therefore had superior and longer therapeutic effects compared to plain hydrogel or MR409.

Several limitations of the study should be noted. First, using only female rats may induce gender bias and efficacy in males warrants further study. Moreover, degeneration and reparative processes of rat coccygeal discs do not fully recapitulate those in human lumbar discs 76. Further studies on large animals, such as goats and monkeys, are needed to confirm the therapeutic effects of MR409. Also, puncture-induced disc degeneration is not comparable to human disc degeneration in duration or mechanism 33. In addition, the ROS-responsive compound requires further improvements to permit long-term function as disc degeneration is a chronic pathology that progresses over years and decades. Also, the mechanisms underlying MR409 efficacy require further research. In view of previous reports 77, we propose that MR409 probably attenuates THBP-induced TRIM16 expression by decreasing cellular ROS via NRF2-dependent pathway.

## Conclusion

We demonstrate that activation of secretory autophagy contributes to disc degeneration in humans and model animals. TRIM16, a cargo receptor in the secretory autophagy pathway, mediates IL-1β secretion under oxidative stress and thus is a potential therapeutic target for controlling IL-1β-mediated pathology. *In vivo* administration of MR409 can suppress secretory autophagy and thereby slow age-related disc degeneration in mice. The intradiscal application of a ROS-responsive MR409-encapsulated hydrogel achieved local controlled release and attenuated needle puncture-induced disc degeneration in rats by inhibiting the secretory autophagy pathway. Inhibition of secretory autophagy is a promising therapeutic strategy against disc degeneration and MR409 is an efficacious candidate compound.

## Supplementary Material

Supplementary figures and tables.

## Abbreviations

ACAN: aggrecan
BSA: Bovine Serum Albumin
DAB: diaminobenzidine
DAPI: 4′,6-diamidino-2-phenylindole
DHI: disc height index
ECM: extracellular matrix
EDTA: ethylenediaminetetraacetic acid
FBS: foetal bovine serum
HE: hematoxylin and eosin
IL-1β: interleukin-1β
MMPs: matrix metalloproteinases
MR: magnetic resonance
NP: nucleus pulposus
PBS: phosphate-buffered saline
PBST: phosphate-buffered saline with Tween
PLGA-PEG-PLGA: poly(lactic-co-glycolic acid)-b-poly(ethylene glycol)-b-poly(lactic-co-glycolic acid)
PI: propidium iodide
PMA: phorbol 12-myristate 13-acetate
ROS: reactive oxygen species
SD: standard deviation
TBHP: tert-butyl hydroperoxide
TBST: Tris-buffered asline with Tween
TRIM16: tripartite motif-containing 16
TUNEL: Terminal deoxynucleotidyl transferase dUTP nick end labeling
μCT: micro-computerized tomography
