# A thermosensitive, reactive oxygen species-responsive, MR409-encapsulated hydrogel ameliorates disc degeneration in rats by inhibiting the secretory autophagy pathway: Erratum

**DOI:** 10.7150/thno.103020

**Published:** 2024-09-12

**Authors:** Qiangqiang Zheng, Haotian Shen, Zongrui Tong, Linxiang Cheng, Yuzi Xu, Zhiyun Feng, Shiyao Liao, Xiaojian Hu, Zongyou Pan, Zhengwei Mao, Yue Wang

**Affiliations:** 1 Spine lab, Department of Orthopedic Surgery, The First Affiliated Hospital, Zhejiang University School of Medicine, Hangzhou 310003, China.; 2 MOE Key Laboratory of Macromolecular Synthesis and Functionalization, Department of Polymer Science and Engineering, Zhejiang University, Hangzhou 310027, China.; 3 Department of Orthopedic Surgery, Zhejiang Provincial People's Hospital, Hangzhou Medical College, Hangzhou 310003, China.; 4 Department of Oral Implantology and Prosthodontics, The Affiliated Stomatology Hospital, Zhejiang University School of Medicine, Hangzhou, 310006, P.R. China.; 5 Dr. Li Dak Sum & Yip Yio Chin Center for Stem Cells and Regenerative Medicine, And Department of Orthopedic Surgery of the Second Affiliated Hospital, Zhejiang University School of Medicine, Hangzhou, China.

This corrects the article “A thermosensitive, reactive oxygen species-responsive, MR409-encapsulated hydrogel ameliorates disc degeneration in rats by inhibiting the secretory autophagy pathway” in Volume 11, Issue 1, 2021, on pages 147-163.

The authors regretfully acknowledge an error in the published version of this article, wherein the MR images for the control and MR409 treatment group in Figure 6B were misused due to labeling errors during the figure compilation process. The corrected version of Figure 6 is presented below.

The authors confirmed that this error did not influence the statistical analysis, the overall results and conclusions of this article. All authors are gratitude to the opportunity to rectify the record. Additionally, the authors make an apology for any inconvenience caused.

## Figures and Tables

**Figure 6:**
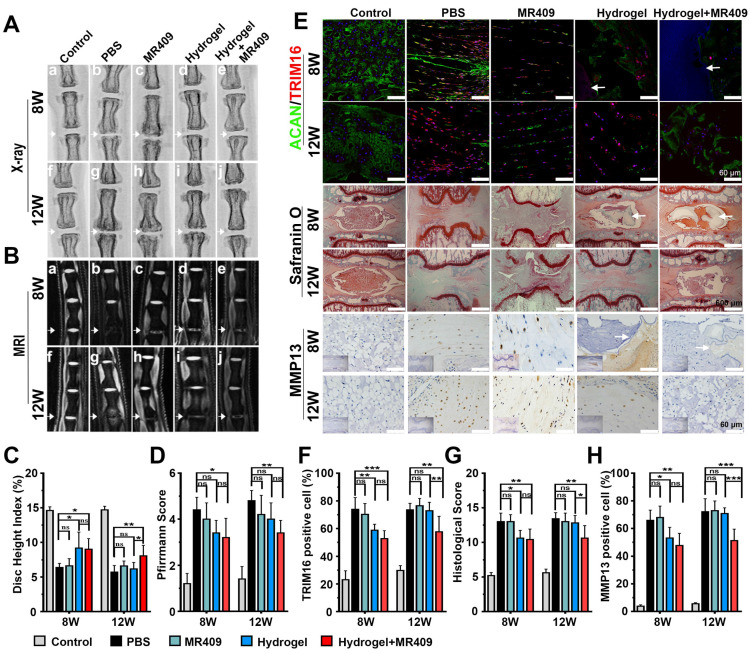
The thermosensitive hydrogel with ROS-responsive MR409-loaded vesicles attenuated needle puncture-induced disc degeneration in rats. (A and B) Representative spine X-ray (A) and MR images (B) from the 5 experimental groups at 8 and 12 weeks. (C and D) Disc height index measured on X-ray images (C) and Pfirrmann score assessed on MR images (D) showed that the MR409-encapsulated hydrogel significantly alleviated disc height loss and attenuated disc degeneration at both 8 and 12 weeks after disc puncture. (E) Representative immunofluorescence staining of ACAN (green) and TRIM16 (red), Safranin O staining, and immunohistochemical staining of MMP13 in experimental discs at postoperative weeks 8 and 12. Arrows indicate residual hydrogel. (F) Reduced numbers of TRIM16-positive cells in discs, indicating that MR409-encapsulated hydrogel inhibited secretory autophagy. (G and H) Histological score (G) and quantitation of MMP13-positive cells (H) showed that MR409-encapsulated hydrogel alleviated disc degeneration at both 8 and 12 weeks after puncture surgery. Data are expressed as the mean + SD of 5 rats per treatment group. Group means were compared by one-way ANOVA with post-hoc Tukey tests. ns, not significant; *p < 0.05; **p < 0.01; ***p < 0.001.

