# Supplementary material for: A thermosensitive, reactive oxygen species-responsive, MR409-encapsulated hydrogel ameliorates disc degeneration in rats by inhibiting the secretory autophagy pathway
Source: Theranostics. 2021 Jan 1;11(1):147–63. doi: 10.7150/thno.47723 (PMC7681093; doi:10.7150/thno.47723)
Supplement: Supplementary file 1 — Supplementary figures and tables. [file thnov11p0147s1.pdf]

Supplementary Information

A thermosensitive, reactive oxygen species-responsive, MR409-encapsulated hydrogel ameliorates disc degeneration through inhibiting the secretory autophagy pathway

Qiangqiang Zheng[1#], Haotian Shen[1#], Zongrui Tong[2], Linxiang Cheng[1], Yuzi Xu[4], Zhiyun Feng[1], Shiyao Liao[3], Xiaojian Hu[1], Zongyou Pan[1,5✉], Zhengwei Mao[2✉], Yue Wang[1✉]

1. Spine lab, Department of Orthopedic Surgery, The First Affiliated Hospital, Zhejiang University School of Medicine, Hangzhou 310003, China
2. MOE Key Laboratory of Macromolecular Synthesis and Functionalization, Department of Polymer Science and Engineering, Zhejiang University, Hangzhou 310027, China
3. Department of Orthopedic Surgery, Zhejiang Provincial People's Hospital, Hangzhou Medical College, Hangzhou 310003, China.
4. Department of Oral Implantology and Prosthodontics, The Affiliated Stomatology Hospital, Zhejiang University School of Medicine, Hangzhou, 310006, P.R. China.
5. Dr. Li Dak Sum & Yip Yio Chin Center for Stem Cells and Regenerative Medicine, And Department of Orthopedic Surgery of the Second Affiliated Hospital, Zhejiang University School of Medicine, Hangzhou, China.

[#]These authors contributed equally to this work.

✉ Corresponding authors:

Yue Wang, Phone: 86-13325710066. E-mail: wangyuespine@zju.edu.cn.

Zhengwei Mao, E-mail: zwmao@zju.edu.cn.

Zongyou Pan, E-mail: panzongyou@zju.edu.cn.

Preparation of PPS-PEG polymer

Synthesis of PEG-Tosyl

A 1.5-g sample of poly(ethylene glycol) methyl ether (Mw ~ 2 kDa) was dissolved in 20 mL of a 1:1 (v:v) tetrahydrofuran (THF)/water mixture, then 4 g of sodium hydroxide was added over an ice bath with vigorous stirring. Excess tosyl chloride (4.5 g) dissolved in 15 mL THF was added drop-wise to the above solution. After overnight reaction under ambient conditions, the solution was extracted three times with dichloromethane and distilled water. The transparent viscous liquid PEG-Tosyl was obtained by removing the solvent.

Synthesis of PEG thioacetate

A 0.9-g sample of PEG-Tosyl was dissolved in 20 mL of dichloromethane and 2.28 g of potassium thioacetate dissolved in 20 mL of a 1:1 dichloromethane/MeOH mixture was added. Following addition of 500 μL trimethylamine, the mixture was incubated for 12 h. The precipitate was filtered and the solvent was removed using a rotavapor. The remaining product was extracted three times with dichloromethane and distilled water. The transparent viscous liquid PEG thioacetate was obtained by removing the solvent.

Synthesis of PEG-PPS diblock polymer

A 200-mg sample of PEG thioacetate was dissolved in 5 mL THF and the solution degassed three times with nitrogen. A 16-mg sample of sodium methoxide in 0.5 mL MeOH was injected into the degassed solution and the mixture left at room temperature for 30 min. Then, 925 mg propylene sulfide was injected into the mixture. After 45 min, 138 mg iodoacetamide was added and the new solution left overnight. The final product was obtained by precipitation with three volumes of ethyl ether.

Supplementary Table1. Patients' disc samples

Characteristics	Degenerated disc group	Non-degenerated
Sample numbers	10	6
Age (years)	56.6 ± 20.1	40.8 ± 7.0
Gender (male/female)	7/ 3	4 / 2
Pfirrmann grade		
I		3
II		3
III		
IV	6	
VI	4	

Note: I–V indicate Pfirrmann grades of discs, listed in Table S5.

Supplementary Table 2. Histological scores of discs.

Histological scores of disc degeneration
I. Cellularity of the annulus fibrosus
Point:
1. Fibroblasts comprise >75% of the cells
2. Neither fibroblasts nor chondrocytes comprise >75% of the cells
3. Chondrocytes comprise >75% of the cells
II. Morphology of the annulus fibrosus
Point:
1. Well-organized collagen lamellae without ruptured or serpentine fibers
2. Inward bulging, ruptured or serpentine fibers in less than one third of the annulus
3.Inward bulging, ruptured or serpentine fibers in more than one third of the annulus
III. Border between the anulusfibrosus and nucleus pulposus
Point:
1. Normal, without any interruption
2. Minimal interruption
3. Moderate or severe interruption
IV. Cellularity of the nucleus pulposus
Point:
1. Normal cellularity with stellar shaped nuclear cells evenly distributed throughout the nucleus
2. Slight decrease in the number of cells with some clustering
3. Moderate or severe decrease (>50%) in the number of cells with all the remaining cells clustered and separated by dense areas of proteoglycans
V. Morphology of the nucleus pulposus
Point:
1. Round, comprising at least half of the disc area in midsagittal sections
2. Rounded or irregularly shaped, comprising one quarter to half of the disc area in midsagittal sections
3. Irregularly shaped, comprising less than one quarter of the disc area in mid sagittal sections

Supplementary Table 3. Sequences of TRIM16 siRNA.

Genes	sense (5′-3′)	antisense (5′-3′)
Si-TRIM16#1	GGUGAACAUCAAACUGCAATT	UUGCAGUUUGAUGUUCACCTT
Si-TRIM16#2	GCAAAUAUUGGACUUCCAATT	UUGGAAGUCCAAUAUUUGCTT
Si-TRIM16#3	CCAUGACUCUGGUUCACAATT	UUGUGAACCAGAGUCAUGGTT

Supplementary Table 4. Primer sequences in real-time PCR

Genes	forward (5′-3′)	reverse (5′-3′)
**Rat SOX9**	GACGT GCAAG CTGGG AAG	GTTGG GCGGC AGGTA TT
**Rat ACAN**	GGCGT CCAAA CCAAC CCGAG	GGAGC TGATC TCATA GCGAT C
**Rat ADAMTS5**	GCTAC TGCAC AGGGA AGAGG	TGCAT ATTTG GAACC CATT
**Rat MMP13**	CCATG ATGGC ACTGC TGA	TGGAG CACAA AGGAG TGGTC TCAA
**Rat iNOS**	TGGGT GAAAG CGGTG TTCTT	TAGCG CTTCC GACTT CCTTG
**Rat COX-2**	TCCAT TTGTG AAGAT TCCTG TGTTG	TCTCA CTGGC TTATG CCGAA A
**Rat TNF-α**	ACCAC GCTCT TCTGT CTACT G	CTTGG TGGTT TGCTA CGAC
**Rat IL-6**	GACTT CCAGC CAGTT GCCTT	GCAGT GGCTG TCAAC AACAT
**Rat GAPDH**	AGACA GCCGC ATCTT CTTGT	CTTGC CGTGG GTAGA GTCAT

Supplementary Table 5. Pfirrmann disc degeneration score

	I	II	III	IV	V
**Structure**	Homogeneous, bright white	Inhomogeneous with or without horizontal bands	Inhomogeneous, gray	Inhomogeneous, gray to black	Inhomogeneous, black
**Distinction of Nucleus and Annulus**	Clear	Clear	Unclear	Lost	Lost
**Signal Intensity**	Hyperintense, isointense to cerebrospinal fluid	Hyperintense, isointense to cerebrospinal fluid	Intermediate	Intermediate to hypointense	Hypointense
**Height of Intervertebral Disc**	Normal	Normal	Normal to slightly decreased	Normal to moderately decreased	Collapsed disc space

Supplementary Table 6. Sense sequences of ATG5 siRNA.

siRNA	Targeting sequences
ATG5 siRNA	5’-GCAACUCUGGAUGGGAUUG-3’

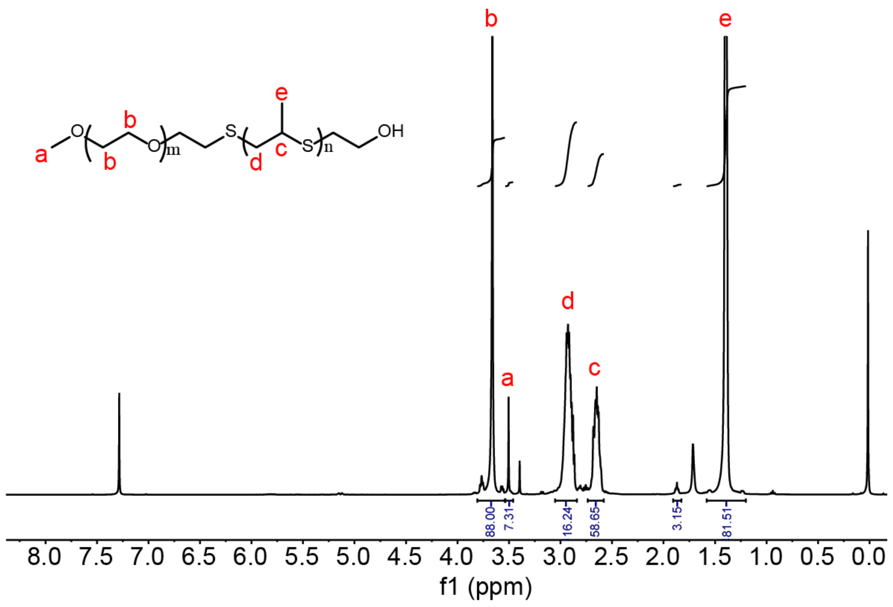

Supplementary Figure 1. ^{1}**H NMR spectrum of the PPS-PEG block polymer.**

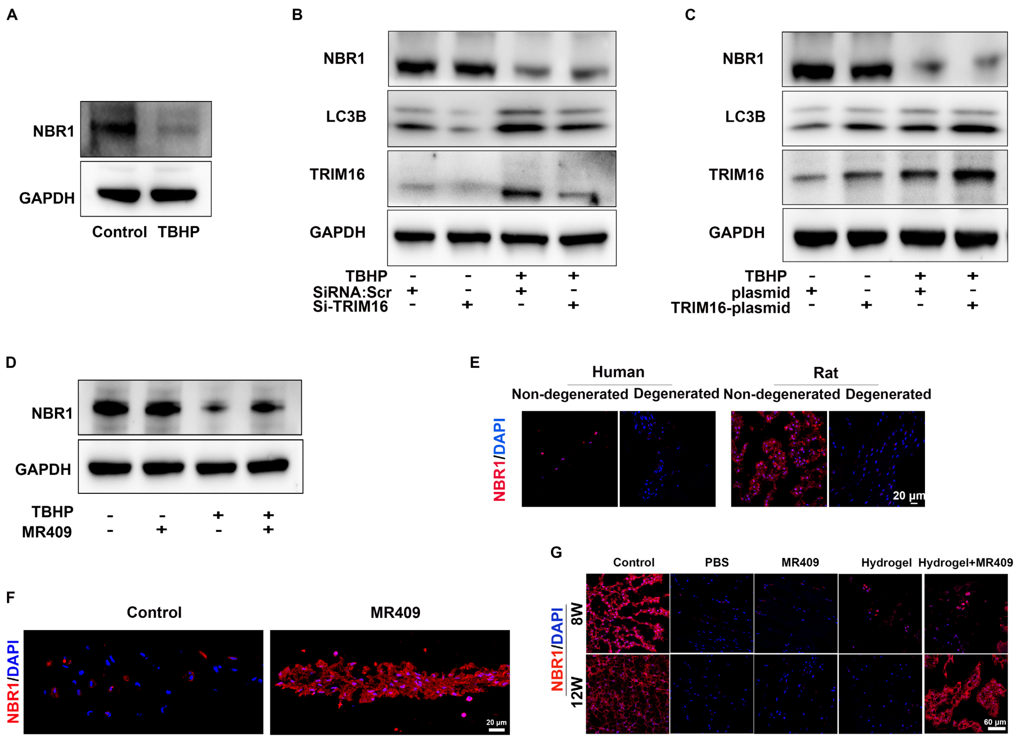

Supplementary Figure 2. (A) Western blot analysis of NBR1 protein expresseion by THP-1 cells after

treatment with vehicle or the oxidant tert-butyl hydroperoxide (TBHP) for 2 h. (**B and C**) Western blot analysis of NBR1, LC3B, and TRIM16 protein expression levels by THP-1 cells transfected with TRIM16 si-RNA or TRIM16-plasmid and then treated with vehicle or TBHP. (**D**) Western blot analysis of NBR1 protein expression by THP-1 cells after treatment with vehicle, MR409, TBHP, or TBHP plus MR409 for 2 h. (**E**) Immunofluorescence analysis of NBR1 expression levels in degenerated and non-degenerated discs of human and rat. (**F**) NBR1 immunofluorescence staining in histological sections of the lumbar disc from 23-month-old mice receiving daily subcutaneous injection of vehicle (control group) or MR409 for 8 months. (**G**) Immunofluorescence staining of NBR1 in rat disc sections obtained from the indicated treatment groups at 8 and 12 weeks.

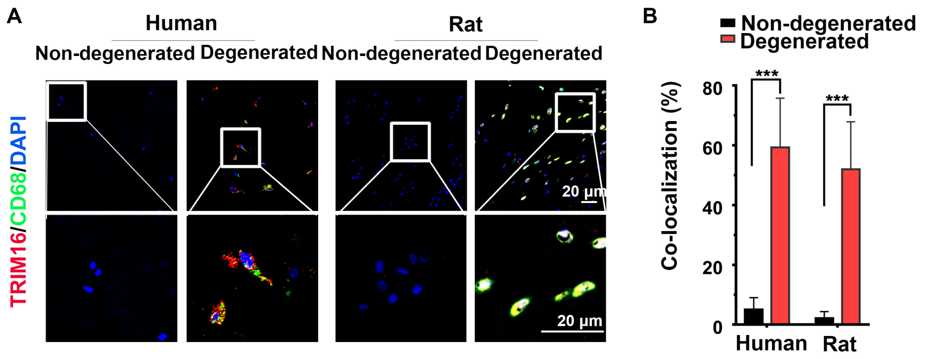

Supplementary Figure 3. (**A**) Immunofluorescence staining of TRIM16 (red) and CD68 (green) in sections from degenerated and non-degenerated discs of human and rat. (**B**) Quantitative analysis of immunostaining.

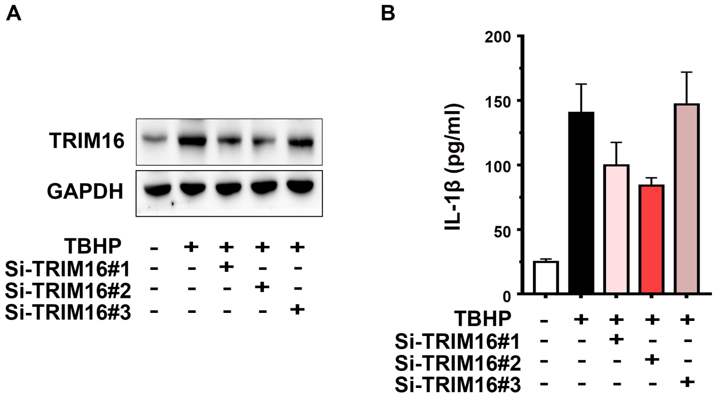

Supplementary Figure 4. (**A**) Immunoblotting assay of TRIM16 protein expression by THP-1 cells transfected with different TRIM16 siRNAs and treated with TBHP. (**B**) ELISA assay of IL-1β in supernatants of THP-1 cells transfected with different TRIM16 siRNAs and treated with TBHP.

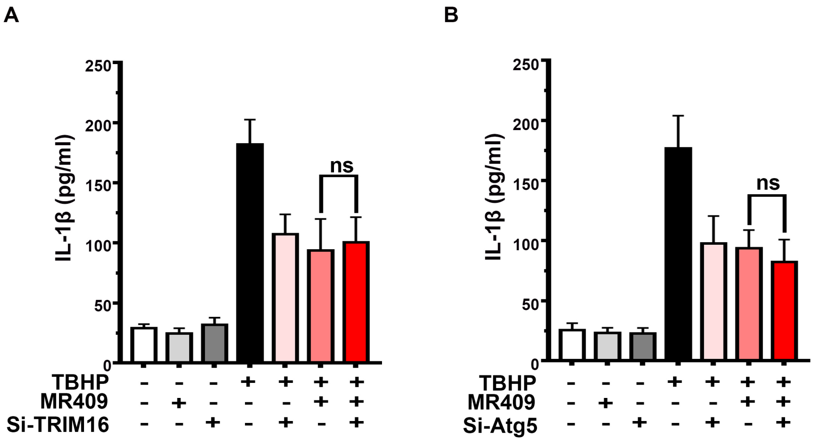

Supplementary Figure 5 A, B. ELISA assay of IL-1β in supernatants of THP-1 cells treated as indicated.

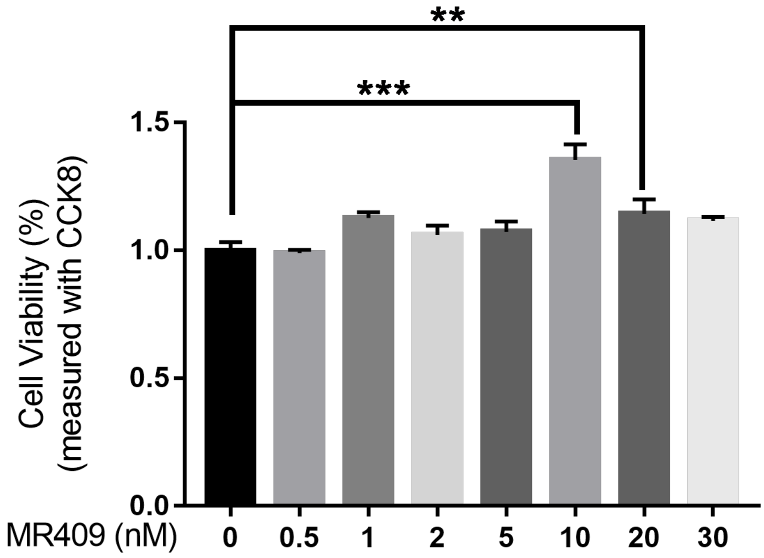

Supplementary Figure 6. nucleus pulposus cells treated with different concentration of MR409 for 24 h.

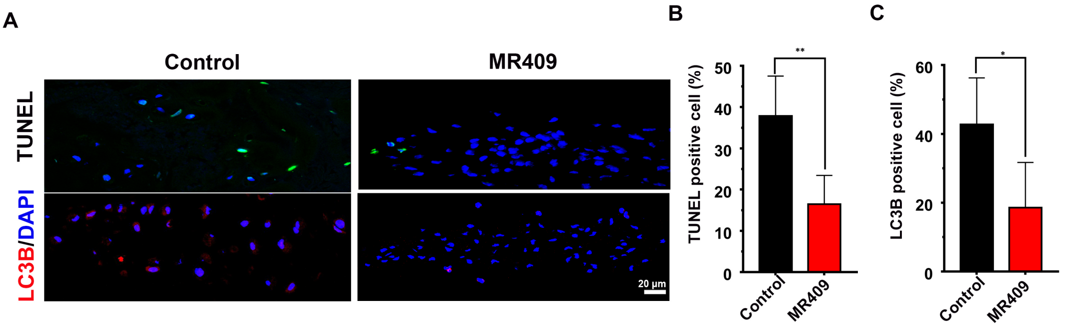

Supplementary Figure 7. (**A**) TUNEL staining of apoptotic cells and immunofluorescence staining of LC3B in lumbar disc sections from 23-month-old mice receiving daily subcutaneous injections of vehicle (control group) or MR409 for 8 months. (**B-C**) Quantitative analysis of immunostaining.

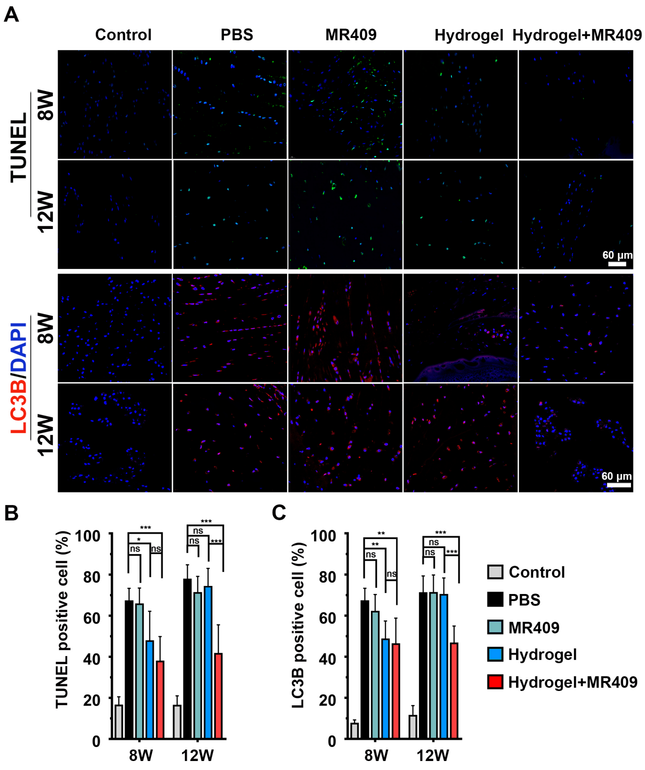

Supplementary Figure 8. (**A**) TUNEL staining of apoptotic cells and immunofluorescent staining of LC3B in rat disc sections from the indicated experimental groups at 8 and 12 weeks. (**B-C**) Quantitative analysis of immunostaining.
